# Brain Metastases from Thyroid Carcinoma: Prognostic Factors and Outcomes

**DOI:** 10.3390/cancers16132371

**Published:** 2024-06-28

**Authors:** Majid Esmaeilzadeh, Oday Atallah, Jörg Andreas Müller, Frank Bengel, Manolis Polemikos, Hans E. Heissler, Joachim K. Krauss

**Affiliations:** 1 Department of Neurosurgery, Hannover Medical School, 30625 Hannover, Germanykrauss.joachim@mh-hannover.de (J.K.K.); 2 Department of Nuclear Medicine, Hannover Medical School, 30625 Hannover, Germany

**Keywords:** thyroid cancer, brain metastasis, surgery, radiotherapy

## Abstract

**Simple Summary:**

In the following study, we aimed to analyze the characteristics, treatment modalities, and outcomes of patients with brain metastases (BMs) from thyroid cancer. To achieve this, from a total of 4320 thyroid cancer patients recorded in our institutional database over a 30-year period, data on 20 patients with brain metastases were retrospectively collected and analyzed. While thyroid cancer patients generally have a favorable prognosis, those with BMs often face a poorer outlook, marked by shorter survival times. Patients who experienced a longer interval (more than 24 months) between the diagnosis of thyroid cancer and the appearance of BMs had significantly better overall survival (OS). Surgical resection, combined with multidisciplinary adjuvant treatment, is essential for managing patients with BMs from thyroid cancer.

**Abstract:**

Intracranial metastases from thyroid cancer are rare. Although the prognosis of thyroid cancer patients is generally favorable, the prognosis of patients with intracranial metastases from thyroid cancer has been considered unfavorable owing to lower survival rates among such patients compared to those without intracranial involvement. Many questions about their management remain unclear. The aim of the present study was to analyze the characteristics, treatment modalities, and outcomes of patients with brain metastases from thyroid cancer. Among 4320 patients with thyroid cancer recorded in our institutional database over a 30-year period, the data of 20 patients with brain metastasis were retrospectively collected and analyzed. The clinical characteristics, histological type of primary cancer and metastatic brain tumor, additional previous distant metastasis, treatment modalities, locations and characteristics on radiologic findings, time interval between the first diagnosis of primary thyroid cancer and brain metastasis, and survival were analyzed. Among our patient cohort, the mean age at initial diagnosis was 59.3 ± 14.1 years, and at the manifestation of diagnosis of cerebral metastasis, the mean age was found to be 64.8 ± 14.9 years. The histological types of primary thyroid cancer were identified as papillary in ten patients, follicular in seven, and poorly differentiated carcinoma in three. The average interval between the diagnosis of thyroid cancer and brain metastasis was 63.4 ± 58.4 months (range: 0–180 months). Ten patients were identified as having a single intracranial lesion, and ten patients were found to have multiple lesions. Surgical resection was primarily performed in fifteen patients, and whole-brain radiotherapy, radiotherapy, or tyrosine kinase inhibitors were applied in the remaining five patients. The overall median survival time was 15 months after the diagnosis of BMs from TC (range: 1–252 months). Patients with thyroid cancer can develop brain metastasis even many years after the diagnosis of the primary tumor. The results of our study demonstrate increased overall survival in patients younger than 60 years of age at the time of diagnosis of brain metastasis. There was no difference in survival between patients with brain metastasis from papillary carcinoma and those with follicular thyroid carcinoma.

## 1. Introduction

Thyroid carcinoma (TC) is the most common malignancy of the endocrine system, with the average age at the time of diagnosis being 40 years of age [1,2,3]. Women are more frequently affected by this malignancy than men, with a male/female ratio of 1/3 being noted in the literature [4,5]. Well-differentiated TC generally have a favorable prognosis with high survival rates. In contrast, poorly differentiated TC are more aggressive and have a poorer prognosis, necessitating more intensive and varied treatment approaches [3,6]. Well-differentiated TC, including papillary thyroid carcinoma (PTC) and follicular thyroid carcinoma (FTC), is the most common histologic type of TC (85% to 90% of all newly diagnosed TC cases), with both types having a favorable prognosis after appropriate treatment [6,7]. Several studies have explored the risk factors for TC patients, which include age, tumor characteristics, genetic mutations, the number of brain metastases, extracranial metastases, and response to treatment [1,7,8]. Distant metastases from TC may involve the lung and bone [8,9]. Brain metastases (BMs) from TC are rare, occurring in only approximately 1% of all patients with TC [10,11]. However, because of the implementation of systematic cerebral imaging before the start of treatment with tyrosine kinase inhibitors (TKIs), a higher frequency of asymptomatic BMs has been documented more recently [12,13,14]. Surgical resection has been favored for the treatment of BMs, either combined with stereotactic radiosurgery (SRS) or whole-brain radiotherapy (WBRT), resulting in the preservation of quality of life and prolonged survival [12,15,16,17,18,19]. Nevertheless, the presence of intracranial metastases from TC has been considered to be associated with poorer outcomes and lower survival rates [20,21,22]. The proper management of TC patients with BMs is challenging because of clinicians’ limited experience with this particular malignancy, and as a result, many questions remain unanswered. Furthermore, there is still a lack of studies in the literature comparing the clinical characteristics and outcomes of patients with BMs from different types of TC.

The aim of the present study was to review our center’s experience with the management of BMs from TC over a 30-year period (Hannover Medical School), with our facility being a maximum care referral center. For this purpose, we analyzed patients’ clinical characteristics, the therapeutic approaches used, and corresponding outcomes. Additionally, we evaluated the prognostic factors affecting cancer-specific survival in patients with BMs secondary to TC.

## 2. Materials and Methods

To identify patients with a diagnosis of BMs from TC, a systematic review of the database of the Department of Neurosurgery and the Department of Nuclear Medicine at Hannover Medical School was performed retrospectively, covering a 30-year period. Among 4320 patients with TC, 20 patients diagnosed with BMs between 1989 and 2019 were identified. Histological confirmation of the thyroid origin of BMs was obtained for 15 patients following surgical resection. The diagnosis of brain metastases in the remaining 5 patients was made through CT or MRI imaging. Charts detailing demographic and clinical data (age, sex, mode of presentation and radiologic features of BMs, treatment modalities including surgery, SRS, WBRT with or without boost, patient outcome, preoperative signs and symptoms, concomitant diseases, and histopathological findings) were evaluated. The progress of treatment over the years in question was taken into account. Neurosurgical procedures were performed according to departmental standard techniques as described previously [23,24]. Patients were followed up until December 2022. The overall survival (OS) after diagnosis of BM was calculated, and the survival time was measured from the diagnosis of BM to the date of death or the last follow-up.

### Statistical Analysis

Cox proportional hazards regression analysis was used for metric predictor variables and categories. In addition, the Cox regression model was applied for survival analysis including the simultaneous effects of multiple risk factors on survival. The model’s coefficients formed the computational basis for hazard ratios (HR) to determine the effect size of predictors on survival (ranging in an open-right interval from 0 to infinity).

As predictors, a list of variables with a possible impact on survival time was selected such as patient age, the time interval between initial diagnosis and the detection of BMs, the number of metastases found, the presence of neurologic deficits, the number of intracranial lesions, and gender. All predictors were categorized (dichotomized) using a two-level nominal scale into set thresholds (Table 1). Survival times are given as months. In the process of censoring the data, four patients with extreme survival time data were excluded. Thus, 80% of the data were found to show survival times between 6 and 77 months. Two predictors, metastases and neurological deficits, showed one missing value each.

## 3. Results

### 3.1. Patient Characteristics

Of the twenty patients with BMs from TC, fourteen were women and six were men (female/male ratio = 2.33:1). The age at the time of diagnosis of TC ranged between 26 and 79 years, with a mean age of 59.3 ± 14.1 years being noted. The mean age at BM diagnosis was 64.8 ± 14.9 years.

### 3.2. Histological Findings of the Primary Tumor

Ten patients were identified as having PTC, seven were identified as having FTC, and three were identified as having a poorly differentiated carcinoma. According to the TMN classification, two patients were in stage I–II: A 63-year-old man with PTC (T2N0M0) underwent total thyroidectomy. Eleven years later, he presented with symptoms including left-sided gait ataxia, dysarthria, left-sided nystagmus, and bilateral dysmetria. CT and MRI scans revealed two lesions (left cerebellum and right occipital). After surgical resection of the cerebellar mass followed by postoperative radiotherapy, despite recommendations for further treatment, the patient declined additional therapy, and died 18 months later. Another patient, a 24-year-old female underwent a total thyroidectomy following a diagnosis of FTC (T2N0M0). Twenty-six months post-surgery, a routine follow-up revealed a mass in the left cerebellum. After surgical resection followed by postoperative radiotherapy, at the last follow-up (138 months later), the patient was alive with no evidence of disease progression. Fifteen patients were in stage III-IV, and in three patients, the stage could not be determined. Nineteen patients presented with other previous and/or synchronous distant metastases: lung (n = 17), bone (n = 7), liver (n = 1), kidney (n = 2), spine (n = 2), and one unknown. Total surgical thyroidectomy was performed in seventeen patients, while three patients underwent subtotal thyroidectomy. Subsequently, 12 patients received a combination of radioactive iodine and radiochemotherapy following total or subtotal thyroidectomy, while one patient was treated with radioactive iodine alone after total thyroidectomy. Lymph node dissection was performed in fifteen patients, and five patients did not undergo lymph node dissection.

### 3.3. Clinical Features of BMs from TC

Table 2 summarizes the demographic and clinical data of the patients with BMs. The mean interval between the diagnosis of TC and BMs was 63.4 ± 58.4 months (range: 0–180 months), and from BM diagnosis to death, the mean interval was 41.2 ± 60.7 months (range 1–252: months). Ten patients were identified as having a single BM, eight were identified as having two or three BMs, and two were identified as having four or more BMs. The presence of BMs was indicated by neurologic symptoms in 11 patients (headache, nausea, motor or sensory deficits, ataxia, aphasia, confusion, and epileptic seizures). The remaining nine patients displayed no symptoms and the BMs were diagnosed incidentally. The diagnosis was made through the use of magnetic resonance imaging in eight patients, computed tomography in six patients, iodine scans in three patients, and PET imaging in three patients. The most common site of brain metastasis was the parietal lobe (10, 32%), followed by the frontal lobe (7, 22%), cerebellum (5, 17%), occipital lobe (5, 17%), temporal lobe (2, 6%), and brainstem (2, 6%). The Karnofsky Performance Score (KPS) in seventeen patients (85%) was >70, and in three patients (15%), it was ≤70.

### 3.4. Treatment

The primary treatment for BMs was surgical resection in fifteen patients, and surgery was the only treatment modality for BMs in four cases. SRS and isotretinoin therapy was used in only one patient. WBRT alone was performed in three patients. The 11 remaining patients underwent a combined treatment regime. Surgical resection coupled with WBRT was performed in eight patients. In one patient, radioactive iodine was applied after surgical resection, and another patient underwent WBRT and tyrosine kinase inhibitor therapy (Lenvatinib) after surgical resection. In another case, WBRT was performed following SRS.

### 3.5. Survival

The patients’ overall median survival time was 15 months after the diagnosis of BMs from TC and ranged from 1 to 252 months. At the time of the last follow-up, sixteen patients had died and four patients were still alive. Information regarding the cause of death was available for 14 patients. Their cause of death was presumed to be related to the progression of multiple BMs, the recurrence of BMs, and the progression of extracranial metastases in twelve patients, while two patients died from septic shock, and in two patients, the cause of death was unknown.

The regression model’s significance level indicated a difference in survival time when at least one of the covariates was included in the model, i.e., the omnibus null hypothesis of multivariate Cox analysis could be soundly rejected (chi2(6) = 17.0149, *p* = 0.0092).

**Figure 1:**
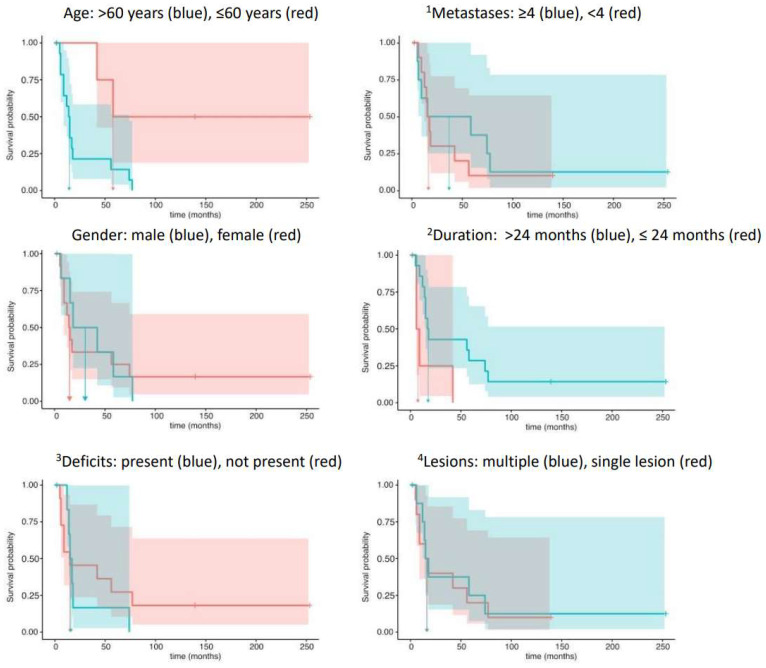
Template of the Kaplan–Meier survival analyses and corresponding 95% confidence intervals of predictors in the Cox regression analyses. **^1^** Number of metastases; **^2^** duration from initial diagnosis to brain metastases; **^3^** neurological deficits; **^4^** number of intracranial lesions.

In detail, the hazard ratio for age, HR = e^Coef^ = 6.19, indicated a highly increased risk of death for patients over 60 years of age. Contrarily, the covariate duration, i.e., an interval longer than 24 months between the initial diagnosis and BM detection, markedly decreased the risk of death (HR = 0.21). Both test results reached statistical significance. The remaining analyses of the covariates metastases (HR = 1.62) and gender (HR = 1.07) showed opposite trends to those of neurological deficits (HR =0.79) and lesions (HR = 0.97) in their statistical relationship with survival times. The four latter effects, however, failed to reach statistical significance (Table 3). Figure 1 displays the Kaplan–Meier survival analyses with corresponding 95% confidence intervals of predictors in the Cox regression analyses.

## 4. Discussion

The prevalence of BMs in patients with TC was rare in our study cohort (less than 1%), which is in keeping with the results of previously published studies [2,13]. BMs from TC, however, have been reported more frequently in the last few years [25,26,27]. This increase is likely due to the more frequent use of cranial imaging and longer survival in patients with advanced disease. BMs were incidentally detected in several of our patients without neurological symptoms following a systemic image survey before targeted therapy. There was no difference between OS in patients with PTC or FTC. However, certain histological subtypes and aggressive variants of PTC, such as the diffuse sclerosing variant, tall cell variant, columnar cell variant, hobnail variant, and solid variant, are associated with a greater likelihood of distant metastases, including to the brain. These variants exhibit more aggressive behavior, higher rates of recurrence, and an increased potential for spread beyond the thyroid gland, which is relevant for both prognosis and treatment planning [7,15]. Patients with a single BM were found to have better outcomes than those with multiple metastases. The results of our study also demonstrate that OS in patients younger than 60 years of age at the time of diagnosis of BM was significantly better compared to patients older than 60 years of age. Additionally, OS was better in patients with a duration longer than 24 months between TC and BM diagnosis.

The interval between the first diagnosis of TC and BM in our study was relatively long, similar to the results of previously published studies [3,16,28,29]. The most common location of BMs was in the telencephalon, while three patients were identified as having cerebellar lesions and two patients were identified as having both infra- and supratentorial BMs. Remarkably, Kwon et al. reported that BMs from lung adenocarcinoma might preferentially involve the distal middle cerebral artery territory and the cerebellum [30]. Furthermore, Kyeong et al. showed that different breast cancer subtypes might show different distributions of BMs [31]. Moreover, Kim et al. postulated that a “seed and soil theory” with the affinity of the tumor to a microenvironment could be partially responsible for the involvement of specific brain regions [3].

The results of our study are in line with previous studies concerning the coexistence of other organ metastases and BMs [2,32,33,34]. In our cohort, 90% of patients had metastases involving two or more organs. The most frequent coexisting metastatic sites were the lung (85%) and bone (35%). Furthermore, the results of our study show that BMs develop generally after the onset of other metastases [2,27].

Treatment decisions for BMs in TC are influenced by various clinical factors including the extent of systemic tumor spread, the specific histopathology type, the tumor’s responsiveness to radioiodine, and its location within the brain [33,34]. As recommended by National Comprehensive Cancer Network (NCCN) guidelines in 2022, surgical removal followed by WBRT or SRS combined with WBRT is recommended for patients with stable systemic disease or those recently diagnosed. Furthermore, either neurosurgical resection or stereotactic SRS is preferred over WBRT for patients who have a solitary BM. In addition, survival was significantly improved by surgical resection of one or more tumor foci. Conversely, WBRT or SRS alone is suggested for patients with multiple (>3) metastatic lesions. According to the results of published studies, radioiodine might not prove effective in treating cranial metastases from thyroid cancer due to the inability of most cranial metastatic lesions to concentrate radioiodine [35,36].

The results of various retrospective studies have shown that the resection of BMs improves OS in patients with BMs from TC [12,37,38]. The results of previous studies [11,39,40] have also shown that patients with a KPS score of 70 or more have a better prognosis with longer survival. Chiu et al. [41] reported that patients with a single BM have better outcomes than patients with multiple BMs; in contrast, Wu et al. [13] reported no difference in patients with a single metastasis in comparison with those with multiple metastases. Genomic alterations in TC, such as BRAF, RAS, RET/PTC, TERT promoter, and TP53 mutations, play a critical role in the disease’s aggressiveness and metastatic behavior, including the development of BM [1,7,15]. Identifying these mutations may help in understanding the prognosis, guiding treatment decisions, and developing targeted therapies to improve outcomes for patients with metastatic TC [21,22].

The results of the present study should be interpreted with caution due to the retrospective design of the study and the inclusion of a sample of patients from a single center. Moreover, the prognostic subgroups defined in the study were not validated in a prospective form, which is not likely to take place, however, owing to the rare incidence of BMs from TC. Furthermore, the diagnostic procedures and therapeutic modalities, both surgical and non-surgical, for treating BM from TC inevitably evolved over the 30-year period. This evolution limits the comparison of the efficacy of different treatment modalities.

## 5. Conclusions

In summary, given their rarity, the exact incidence of BMs from TC has been difficult to estimate. Although patients with thyroid cancer generally have a favorable prognosis, those with BMs typically experience a poorer prognosis, characterized by a shorter survival period. In our study, the number of BMs did not play a significant role in determining patient survival. Furthermore, in the case of a longer interval (longer than 24 months) between TC diagnosis and BM manifestation, patients have significantly better OS. Both surgical resection and multidisciplinary adjuvant treatment are critical for patients with BMs from TC.

## Figures and Tables

**Table 1:** Set of predictors for the Cox regression model.

Predictor	Meaning	Threshold	Unit
Age	Patients’ age	≤60, >60	Years
Duration	Time interval between the initial and brain metastasis diagnostics	≤24, >24	Months
Metastases	Number of metastases found	≤4, >4	
Neurological deficits	Existence of neurological deficits	Yes or no	
Gender	Patients’ gender	Male or female	
Lesions	Number of intracranial lesions	Single or multiple	

Statistical analyses were performed using JMP^®^, Version 16.2.0 (SAS Institute Inc., Cary, NC, USA, 1989–2021) and R Core Team (ver. 4.2.3, R Foundation for Statistical Computing, Vienna, Austria, 2023). A *p*-value of less than 0.05 was considered statistically significant.

**Table 2:** Demographic and clinical characteristics at the time of BM diagnosis.

	N
Mean age at BM diagnosis (years)	64.8 (26–89)
Mean interval between initial diagnosis and BM diagnosis (months)	63.4 ± 58.4
Mean interval between BM diagnosis and death (months)	41.2 ± 60.7
Symptoms associated with BMs	
Yes	11
No	9
Modality of diagnosis	
MRI	8
CT	6
FDG-PET-CT	3
Iodine-131 whole-body scintigraphy	3
KPSS	
>70%	17
≤70%	3
Number of intracranial lesions	
Single	10
Multiple	10
Number of distant metastases	
≤3	11
>3	8
Unknown	1
Treatment	
Surgery	15
Radiosurgery	1
WBRT	3
Isotretinoin	1
Postoperative adjuvant therapy	
WBRT	10
RCT	1
Radioactive iodine	1
WBRT + TKI	1
WBRT + Isotretinoin	1
None	6

WBRT: Whole-brain radiation therapy; RCT: radiochemotherapy; TKI: tyrosine kinase inhibitor; KPS: Karnofsky Performance Score. MRI: magnetic resonance imaging, CT: computed tomography, FDP-PET-CT: fluorodeoxyglucose–positron emission tomography–computed tomography, BM: brain metastasis.

**Table 3:** Multivariate analysis of covariates.

Covariate	Coef	HR	95% CI_coef_	LR Test	*p*-Value	Unit
Age (>60)	1.8228	6.1892	0.6865, 3.5655	chi2(1) = 12.0981	0.0005	Years
Duration (>24)	−1.5682	0.2084	−2.8077, −0.5466	chi2(1) = 9.2470	0.0024	Months
Metastases (<4)	0.4846	1.6235	−0.1578, 1.2459	chi2(1) = 2.1383	0.1437	
Neurological deficits (no.)	−0.2417	0.7853	−1.1459, 0.7033	chi2(1) = 0.2721	0.6020	
Gender (female)	0.0712	1.0738	−0.5441, 0.7342	chi2(1) = 0.0501	0.8229	
Lesions (multiple)	−0.0267	0.9737	−0.7675, 0.7816	chi2(1) = 0.0048	0.9450	

HR: hazard ratio.

## Data Availability

The data presented in the following study are available from the corresponding authors upon request.
